# Clustered Cardiometabolic Risk and the “Fat but Fit Paradox” in Adolescents: Cross-Sectional Study

**DOI:** 10.3390/nu16050606

**Published:** 2024-02-22

**Authors:** Noelia González-Gálvez, Ana Belén López-Martínez, Abraham López-Vivancos

**Affiliations:** Facultad del Deporte, UCAM Universidad Católica de Murcia, 30107 Murcia, Spain; ngonzalez@ucam.edu (N.G.-G.); alvivancos@ucam.edu (A.L.-V.)

**Keywords:** overweight, obesity, fitness, cardiorespiratory, children, school, blood pressure, diabetes, cholesterol

## Abstract

The “fat but fit paradox” states that people who are fit have a lower cluster cardiometabolic risk (CCMR), even if they are overweight or obese. Therefore, the objective was to investigate the CCMR between four categories based on the “fat but fit paradox” variable, in different fitness categories—cardiorespiratory fitness, muscular fitness, and physical fitness—in adolescents. Body composition, cardiorespiratory fitness, muscle fitness, blood samples, and blood pressure were assessed in 230 adolescents, and cardiometabolic risk and three different “fat but fit paradox” variables were calculated. Participants with a higher CRF exhibited a lower CCMR within their body mass index (BMI) category (*p* < 0.05). Participants with a high BMI and high muscular fitness showed a lower CCMR than participants with a low muscular fitness and a similar BMI, or low BMI and low muscular fitness (*p* < 0.05). When both variables, CRF and muscular fitness, were combined, their effectabove CCMR increased (*p* < 0.05). Across all fitness categories, the fat and unfit group, whether considered individually or combined, exhibited the highest risk of CCMR (*p* < 0.05). This study confirms the “fat but fit paradox” in different physical fitness categories, showing the importance of both CRF and muscular fitness as predictors of CCMR, with the combination of both variables showing a greater agreement.

## 1. Introduction

Childhood obesity has become a global epidemic, making it one of the biggest public health challenges in the 21st century. The World Health Organization states that the rate of childhood obesity has increased significantly in recent decades. This gradual increase in the prevalence of childhood obesity is not only a concern on its own, but it also has serious consequences for the long-term health of those affected [1,2]. Numerous epidemiological studies have established a direct relationship between excess weight in childhood and the early onset of cardiovascular diseases, insulin resistance, type 2 diabetes, dyslipidemia, and other cardiometabolic risk factors [3,4,5]. On the other hand, the autonomic nervous system also plays a crucial role in regulating eating behavior. Taking into account the thermoregulatory function of food intake, the regulation of body temperature is strictly related to the control of body weight. Peptides and hormones, such as orexins and adiponectin, which are involved in temperature control, the regulation of locomotor activity, and food intake, influence obesity-related diseases [6]. Childhood obesity has been identified as a significant risk factor for the development of cardiometabolic disorders at an early age, and their persistence into adulthood [7].

This contributes towards an increase in disease and the likelihood of premature mortality in adult life [8]. Obesity, defined as excess fatty tissue, is widespread around the world and affects all age groups [3]. Obesity, among other factors, is a well-known risk factor for metabolic syndrome, which comprises cardiovascular diseases, diabetes mellitus, hypertension, and atherosclerosis, as well as other complications. In this sense, different researchers have focused on the analysis of the cluster cardiometabolic risk (CCMR) [9,10].

The “fat but fit paradox” is a theoretical paradigm based on evidence that emerged in the late 1990s, which suggested that moderate-to-high levels of physical fitness, mainly cardiorespiratory fitness (CRF), could counteract the adverse influence of obesity on cardiometabolic risk [11,12]. This phenomenon implies that individuals who are overweight or obese but maintain good levels of CRF, could show better health outcomes as compared to sedentary overweight or obese individuals. This paradox is observed in children, adolescents, adults, and older adults [12].

Some research studies have demonstrated the combined association between weight and CRF and mortality. Individuals with a worse CRF, regardless of their weight, showed higher risks of death [13]. Other studies indicate that CRF shows a higher mortality predictive role than obesity, and that physical fitness levels significantly reduce the risk of mortality regardless of the individual’s body mass [14]. It has been shown that greater physical activity for maintaining better levels of CRF has proven to be an effective and safe method for the primary and secondary prevention of diseases in all weight groups [15].

Several studies [11,15,16,17] have established that with body mass index (BMI) as a mediating variable, the relationship between CRF and cardiometabolic risk completely disappears in the case of girls and is significantly attenuated in boys. These results do not fully support the “fat but fit” paradigm, which postulates that a person with excess body fat but with a high level of CRF (the fat–fit phenotype) has a more favorable cardiometabolic profile than a person with excess body fat but a low level of CRF. Other studies have demonstrated an inverse relationship between physical fitness and cardiometabolic risk factors in children, suggesting that higher levels of physical fitness correlate with a reduction in the prevalence of obesity and an improvement in cardiometabolic markers [18]. In addition, other scientific evidence suggests that moderate-to-high CRF could counteract the negative effects of obesity, especially in children and adolescents [19].

Another fitness variable that has been widely and independently investigated is strength. Numerous studies classify strength as a cardiovascular risk factor, and indicate that it is linked to mortality [12,20,21]. However, some studies that investigated the “fat but fit paradox” did not usually include this parameter [5,22]. Therefore, it is not known whether this parameter for physical condition affects this paradox, or whether other variables should be considered.

For this reason, it is deemed necessary to elucidate the effect of the different physical condition variables relevant to health, CRF and strength, both individually and in combination. Therefore, the objective of the present research is to investigate CCMR and different individual risk factors for cardiometabolic risk scores between four categories based on the “fat but fit paradox” variable for different fitness categories—CRF, muscular fitness, and physical fitness—in adolescents. The present research posits the hypothesis that the “fat but fit paradox” variable in different fitness categories—CRF, muscular fitness, and physical fitness—in adolescents, will be confirmed regarding its influence on CCMR and the individual risk factor of a cardiometabolic risk score between four categories according to the variable. Sedentary subjects will showa greater cardiometabolic risk within their weight category both for CRF and for muscular fitness and physical fitness (CRF and muscular fitness).

## 2. Materials and Methods

### 2.1. Study Design

This multicenter cross-sectional study was carried out in a secondary school in Murcia, Spain. The participants were aged between 12 and 13 years old. The inclusion criteria were (a) being physically active in physical education sessions; (b) agreeing to participate in the study and having the parent/guardian and adolescent sign the consent form; and (c) being present at the time of the assessments. The exclusion criteria were presenting any musculoskeletal, neurological, cardiological, metabolic, or rheumatic pathologies. A total of 230 adolescents were recruited, who had a mean age of 12.19 ± 0.49. Of these, 104 participants were male (45.2%), while 126 were female (54.8%). The participants showed a mean height of 152.71 ± 8.29 cm, a mean weight of 48.72 ± 12.56 kg, and a mean BMI of 20.69 ± 3.98 kg/m^2^. The trial design was registered at ClinicalTrial.gov (Code: NCT05544370) and followed the STrengthening the Reporting of OBservational studies in Epidemiology (STROBE) Statement. The ethical approval for this study was obtained from the Universidad Católica de Murcia (CE061914) and was implemented according to the guidelines for human research from the Helsinki Declaration.

### 2.2. Assessment

The same trained researchers used standardized conditions to measure the variables. Blood samples, blood pressure, anthropometry, and body composition, in that order, were assessed in the laboratory set at a standardized temperature of 24 °C. The participants were instructed to wear light clothing, shorts, and a t-shirt.

Subsequently, the physical condition tests were randomly evaluated. The participants wore comfortable sports clothing and sports shoes. Before the measurements, the participants did not perform any warm-up or stretching exercises, and a 5-min rest period was provided between tests. Each test is explained below.

Anthropometry and body composition assessments were performed. Body mass was determined using a portable digital scale (TANITA BF-522 W, Tokyo, Japan), while height was measured using a SECA 217 stadiometer (SECA, Hamburg, Germany) with an accuracy of 0.1 cm. BMI was calculated using the following formula: body mass (kg)/squared stretch stature (m^2^). Fat mass (FM) percentage was gauged using the same portable digital scale (TANITA BF-522 W, Tokyo, Japan) with an accuracy of 0.1 cm. Waist circumference (WC) was measured with a kinanthropometry tape with an accuracy of 0.1 cm. The WC and the anthropometric measurements were taken in accordance with the guidelines set by the International Society for Advancement of Kinanthropometry [23].

CRF was evaluated using a progressive running test, “the 20-Shuttle run test” [20]. In this test, the participants run back and forth across a 20 m course, synchronizing their crossing of the line with a sound signal from a pre-recorded tape. The initial speed was set at 8.5 km/h, increasing by 0.5 km/h every minute. The test concluded when a participant could no longer maintain the specified speed according to the pre-recorded tape. The last stage number announced was recorded, and the corresponding running speed was determined from reference tables. The maximum oxygen consumption (Vo2max, mL/kg/min) was estimated based on the number of laps completed by the participants, using the equation devised by Leger et al. (Vo2 max = 31.025 + 3.238 X − 3.248 A + 0.1536 A X; where X = running speed and A = age) [24].

Muscular fitness was assessed through two different tests: the handgrip test and the standing long jump test. The handgrip test involved participants standing with arms at their sides. To familiarize themselves with the device and the test, each participant executed one repetition with each hand. Subsequently, they were instructed to exert maximal strength for a duration of 3 s with their right hand. The highest peak strength (kg) observed between the three attempts was selected for the analysis. A digital grip strength dynamometer (TKK 5401; Takei Scientific Instruments Co., Ltd., Tokyo, Japan) with an accuracy of 0.1 kg was utilized for this measurement. To account for variations in body size, handgrip strength was normalized by body weight (kg) [25]. Lower body strength was evaluated from a standing position with the feet positioned roughly shoulder-width apart. The adolescent executed a forward jump, aiming to cover as much distance as possible. The test was conducted twice, and the longest distance attained during the jumps was documented in centimeters [26] with a tape with an accuracy of 0.1 cm. The values of the handgrip and standing long jump tests were converted into z-scores. The muscular fitness score was calculated using the following formula: handgrip strength/body weight (z-score) + standing long jump (z-score) [22].

Physical fitness was calculated with the following: Course Navette test (z-score) + handgrip/body weight (z-score) + standing long jump (z-score). Physical fitness values, individual and combined with CRF, muscular fitness, or physical fitness, were categorized into four groups using the mean of the data: high physical fitness (z-score) (>50th percentile) and low physical fitness (z-score) (≤50th percentile) [27].

The “fat but fit paradox” variable was calculated by utilizing the categories of high and low BMI and high and low physical fitness (both individually and combined). Four distinct groups were established: unfat + fit (UF), unfat + unfit (UU), fat + fit (FF), fat +unfit (FU) [22].

Blood samples were obtained through finger punctures between 8:00 and 9:00 a.m. The Afinion™ Analyzer (Alere, Ltd., Stockport, UK) was employed for the lipid panel test. This test measured various lipid components such as low-density lipoprotein cholesterol (LDL), high-density lipoprotein cholesterol (HDL), non-HDL lipids (LipidNonHDL), and triglycerides (Trig) in whole blood, serum, and plasma. These measurements were utilized for the diagnosis and treatment of lipid disorders. The glucose test was performed using the Accutrend sensor (Roche Diagnostics, Mannheim, Germany). This machine measures with an accuracy of 1 mg/dL.

A Colin BP 880 automatic device from Critikron, Inc. in Tampa, FL, USA, with an accuracy of 1 mm Hg was used to gauge systolic blood pressure (SBP) and diastolic blood pressure (DBP). The measurements were performed on the left arm, repeated twice at 5-min intervals, while the participant was seated. Mean blood pressure (MBP) was determined using the following formula: 1/3 (SBP-DBP) + DBP. MBP is commonly utilized in clinical settings and evaluation protocols, and in a prior study as a mediating variable, as it enables blood pressure to be represented as a single variable [28].

CCMR was determined by computing a sex and age-specific z-score for WC, triglycerides-to-HDL ratio (TGs/HDL), fasting glucose (FG), and mean blood pressure (MBP). The CCMR index was presented as a continuous variable, representing the average z-score across the four cardiometabolic risk measurements. Additionally, adolescents were classified as having an elevated CCMR if their CCMR index exceeded one standard deviation above the mean [12].

### 2.3. Statistical Analysis

The normal distribution of the data was assessed through the Kolmogorov–Smirnov test, and Mauchly’s W-test was used to evaluate the sphericity of the data. A descriptive analysis was conducted for the quantitative variables, with the results shown as means and standard deviations, while the qualitative variables were summarized with sums and percentages. ANCOVAs were performed to assess the mean differences in cardiometabolic risk (z-score) and the individual cardiometabolic risk factors associated with the combined “fat but fit paradox” variable. Similarly, post hoc analyses were used to examine the mean differences between groups with adjustments of confidence intervals using the Bonferroni test. The likelihood of having a high cardiometabolic risk in relation to various classifications of the “fat but fit paradox” variable (both individual and combined) was examined through binary logistic regression analyses.

The statistical analysis was carried out using IBM SPSS Statistics (version 24.0) with a significance level set at *p* < 0.05.

## 3. Results

### 3.1. “Fat but Fit Paradox” and Cardiometabolic Risk

**Figure 1:**
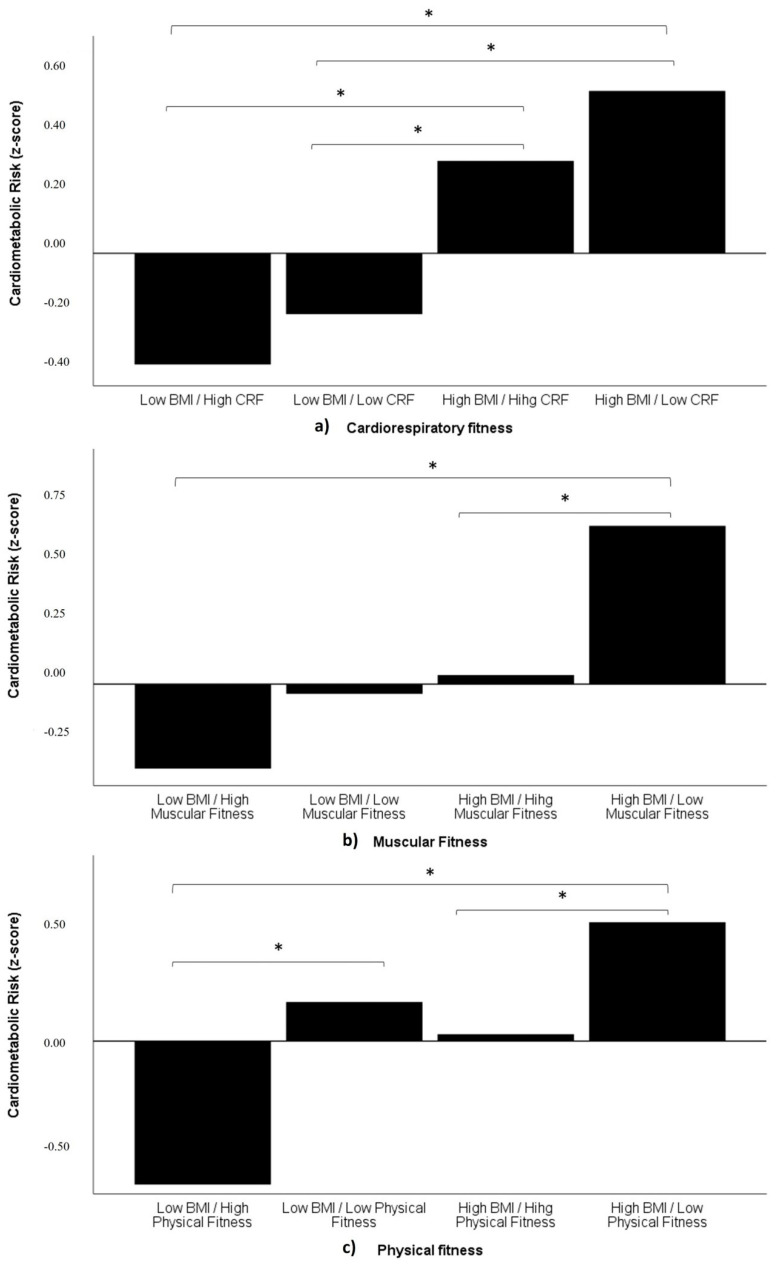
Differences of cardiometabolic risk score between different fitness categories: (**a**) CRF, (**b**) muscular fitness, and (**c**) physical fitness. * = *p* < 0.05.

Figure 1 depicts different cardiometabolic risk scores in relation to the “fat but fit paradox” variable, BMI, and various fitness categories (CRF, muscular fitness, and physical fitness).

Participants with a higher CRF exhibited a significantly lower cardiometabolic risk within their BMI category (UF vs. UU; FF vs. FU) (Figure 1a). This suggests that despite BMI being more influential than CRF, the latter acts as a protective factor against cardiometabolic risk.

Considering the muscular fitness categories, a non-significant increase in cardiometabolic risk was observed. The UF group displayed the lowest risk, with participants in the UU and FF groups showing similar risk levels, while those in the FU group exhibited a higher cardiometabolic risk than the FF group (Figure 1b).

When combining CRF and muscular fitness into the physical fitness category, the results indicated that the participants in the UF group have a significantly lower cardiometabolic risk as compared to those in the UU and FU groups. This suggests that a low physical fitness is associated with an increased cardiovascular risk independent of BMI. Conversely, FF group participants did not show significant differences from the UF or UU group participants, although a significant difference was observed with the FU group. This indicates that despite having a high BMI, if physical fitness is high, the cardiometabolic risk is similar to that of an individual with a normal BMI but not fit, and lower than an individual with a high BMI and lower physical fitness (Figure 1c).

### 3.2. Odds of Cardiometabolic Risk and “Fat but Fit Paradox” and Odds Ratio of Cardiometabolic Risk

Table 1 displays the odds ratio analysis of cardiovascular risk between different physical fitness categories and BMI. Across all fitness categories (CRF, muscular fitness, and physical fitness), the FU group, whether considered individually or in combination, exhibited the highest risk of cardiometabolic risk, significantly. Conversely, the UF group, whether considered individually or in combination, showed the lowest risk of cardiometabolic risk, with a 96.75% (CRF), 95.2% (muscular fitness), and 99.9% (physical fitness) lower risk than the FU group. Similarly, the FF group, whether considered individually or in combination, showed a 57.3% (CRF), 93.9% (muscular fitness), and 95% (physical fitness) lower cardiovascular risk than the FU group.

### 3.3. Individual Metabolic Risk and “Fat but Fit Paradox”

Table 2 presents the means and standard deviations of the unfat/fit (UF), unfat/unfit (UU), fat/fit (FF), and fat/unfit (FU) groups. In the CRF category, the FF and FU groups exhibited significantly higher BMI, WC, and fat mass (FM) values than the UF and UU groups. Additionally, the FU group showed significantly lower BMI, WC, and FM values than the FF group. The FF group had a significant lower HDL as compared to the UF and UU groups, with no difference between the FF group and the unfat groups.

Regarding the muscular fitness category, no differences were observed in BMI between the UF and FF groups, while the FU group had a higher BMI than the FF group. With respect to WC, within the unfat group, those with a higher muscular fitness showed a lower WC than those with a lower muscular fitness. The same association was observed in the fat groups.

For the physical fitness category, there was no difference in BMI between the UF and FF groups, while the FU group had a higher BMI than the FF group. Regarding WC, the FU group had a significantly larger waist circumference than the unfat groups; however, this difference was not present between the FF group and the unfat groups. Lastly, for HDL, only the UF group showed a higher HDL compared to the FU group with no differences among the other groups.

## 4. Discussion

The main finding of the study was that the FU group showed a significantly higher cardiometabolic risk regardless of the variable considered (CRF, muscular fitness, and physical fitness). Likewise, it was shown that within the group considered fat, those with a higher cardiorespiratory fitness, muscular fitness, or physical fitness presented a significantly lower cardiometabolic risk than those with a low CRF, muscular fitness, or physical fitness. This aspect highlights the relevance of BMI, CRF, and muscular fitness, as it has been shown that overweight and obesity in children is associated with metabolic syndrome in adults [29]. For this reason, preventing overweight and obesity at an early age is necessary [30]. Furthermore, overweight and obesity are the main cardiometabolic risk factors in children and adolescents [31].

Obesity has traditionally been related to cardiorespiratory capacity [12,28,32,33,34,35]. In addition, both variables are individually associated with waist circumference and fat mass, and with LDL [12,32,34]; HDL [1,32,36], triglycerides [12,32,36]; MBP [15,28,32], and CCMR [12,32,34].

On the other hand, muscular strength has also been shown to be related to cardiovascular diseases in young adults [12,20,21], and it has been associated with a greater risk of metabolic syndrome [19,37].

When considering the “fat but fit paradox” in the cardiovascular fitness variable, the participants considered to have a low BMI and low cardiovascular fitness showed a significantly lower cardiometabolic risk than participants with a high BMI and high cardiovascular fitness. Similar results have also been described in previous research [22]. However, the present study included a risk analysis that indicated a significantly and increasing risk of presenting cardiovascular risk when comparing the FF, UU, and UF groups with the FU one, respectively. In this sense, some researchers have indicated that the “fat but fit paradox” is true for children and adolescents who are overweight and obese, although not in children who have a normal weight [38]. Another study did not find significant differences between the UU and FF groups, assuming cardiovascular fitness as a protective variable against cardiovascular risk [27,39].

When the muscular fitness variable is taken into account in the “fat but fit paradox” variable, the results changed only slightly. Children and adolescents with a low weight and low muscular fitness showed the same cardiovascular risk as children and adolescents with a high weight and high muscular fitness; and a difference in risk was observed between overweight and obese participants according to their muscular fitness. Likewise, after the risk analysis, a significantly and increasingly greater risk was observed when comparing FF, UU, and UF with FU, respectively. This highlights the relevance of muscular fitness on cardiovascular risk. In this sense, previous research has shown a significantly lower risk in the high BMI and high muscular fitness group compared to the high BMI and low muscular fitness group, as well as an increased risk of developing cardiovascular risk [22]. Likewise, a greater fat mass and lower muscular strength have been associated with a greater cardiometabolic risk in young adults, indicating that both variables are independently and jointly associated with a greater cardiometabolic risk. [5].

Muscular strength has been described as an emerging risk factor for cardiovascular disease, as well as a cause of death, in young adults [12,20,21]. Thus, muscular fitness has been associated with metabolic syndrome and lipid metabolism in young people [37,40], promoting insulin sensitivity and glycemic control [41,42] and reducing adipose tissue [5]. In a longitudinal study, it was shown that strength in childhood was linked to metabolic syndrome in adults. In this sense, in the present study, the variable handgrip strength test and the standing long jump were used to determine the muscular fitness variable, which is normalized in relation with the body weight; these variables are described as having a greater power to discriminate cardiometabolic risk within the different muscular fitness tests [43].

The last analysis of the present study combined cardiovascular fitness and muscular fitness, which is described as physical fitness in the “fat but fit paradox” variable. In this case, it is shown that this paradox is true in both low weight and high weight groups, with the low physical fitness group showing a greater cardiovascular risk than the high physical fitness group. Likewise, there was no difference in cardiovascular risk between the UU and FF groups in addition to showing, as in the previous cases, a significantly and increasingly greater risk when comparing FF, UU, and UF, with FU, respectively. These data point out the relevance of both variables as protectors of cardiovascular risk, and that the combination of both will have a greater protective effect against cardiovascular risk. This suggests that not only should cardiovascular work programs be prescribed for overweight and obese children and adolescents, but they should also include strength work activities that could be more attractive for this population [22,43,44]. In this sense, the present study supports global recommendations for the practice of physical activity, which includes performing 60 min of moderate to vigorous physical activity daily in addition to incorporating strength work activities in children at least three times a week [45]. On the other hand, some authors highlight that strength-endurance activity work seems to be more relevant than maximum muscular strength when associated with cardiometabolic risk [46].

The present study presents an innovative approach, as it includes strength as a fitness variable in addition to cardiovascular capacity. Traditionally, the variable cardiovascular fitness has been used in the “fat but fit paradox”, and only a few studies have included strength as an individual variable or together with cardiorespiratory capacity, shaping a new category of physical fitness [11]. On the other hand, another strength of the present study is the analysis of cardiovascular risk through continuous variables instead of expressing the prevalence according to a MetS dichotomous variable. A continuous score provides more information about health status and risk factors and is easily calculable. [47]. In this sense, the use of cluster cardiometabolic risk, instead of the sum of each of the cardiometabolic risks, is more useful for the early detection of cardioembolic risk [32,38]. Another strength of the present study was the calculation of the BMI z-score, CRF z-score, handgrip z-score, standing long jump z-score, and cardiometabolic risk, according to age and sex, as in previous studies [11,19,32].

The present study is not without limitations. One of the limitations is the use of BMI as a variable for defining overweight and obesity. Although all studies that analyze the “fat but fit” paradox have used this variable, it does not differentiate body composition and does not discriminate between fat mass and muscle mass. Therefore, its use may not be valid for part of the population that has muscle mass values above or below the average, and it may overestimate or underestimate overweight or obesity. The use of fat mass for the analysis of this paradox could be more suitable [32]. On the other hand, in the present study, the maturational state of children and adolescents was not taken into account. The maturational state could influence body composition, CRF, and muscular fitness [48,49], and although the sample that participated in this study showed mean values of BMI, CRF, and muscular fitness similar to the average for their age, as shown in previous studies [50], the present research cannot be extrapolated to other groups. On the other hand, the analysis of psychological well-being should also be considered in this type of research [51]. In this sense, the analysis of the “fat but fit paradox”, taking into account variables such as fat mass, as well as the maturational state of children and adolescents, is considered for future studies.

## 5. Conclusions

This study confirms the “fit but fat paradox” of different physical fitness categories, showing the importance of both CRF and muscular fitness as predictors of cardiovascular risk with the combination of both variables showing a greater agreement.

The present results are considered relevant from a clinical and public health point of view. These findings highlight the relevance of not exclusively focusing on maintaining a weight below that of overweight and obesity but also improving or maintaining good cardiorespiratory capacity and muscular fitness as a protection against developing a cardiometabolic risk in childhood as well as the relevance of increasing the number of hours of physical education at school [52].

## Figures and Tables

**Table 1:** Odds ratio of cardiometabolic risk between different physical fitness categories and BMI.

	CRF		Muscular Fitness		Physical Fitness	
	OR	*p*-Value	OR	*p*-Value	OR	*p*-Value
UF	0.0325	<0.001	0.048	<0.001	0.001	<0.001
UU	0.096	<0.001	0.051	<0.001	0.083	<0.001
FF	0.427	0.048	0.061	<0.001	0.05	0.049
FU	1		1		1	

Legend: FU = fat unfit; UU = unfat unfit; FF = fat fit; UF = unfat fit.

**Table 2:** ANCOVA model of cardiometabolic risk factor by “fat but fit paradox” categories.

	UF	UU	FF	FU	F	*p* Value	Pairwise Comparisons					
							1–2	1–3	1–4	2–3	2–4	3–4
**Cardiorespiratory fitness**												
BMI (kg/m^2^)	17.97 ± 1.55	18.18 ± 1.55	23.88 ± 2.37	25.34 ± 3.75	149.25	<0.001		<0.001	<0.001	<0.001	<0.001	0.016
WC (cm)	63.91 ± 5.10	64.45 ± 6.02	78.33 ± 7.21	83.90 ± 10.40	107.19	<0.001		<0.001	<0.001	<0.001	<0.001	0.002
FM (%)	16.68 ± 4.65	19.35 ± 5.53	28.41 ± 5.67	31.65 ± 7.29	103.70	<0.001		<0.001	<0.001	<0.001	<0.001	0.009
LDL (mg/dL)	80.18 ± 20.31	86.31 ± 25.30	82.77 ± 22.96	96.88 ± 23.69	2.98	0.033						
HDL (mg/dL)	53.11 ± 17.74	53.04 ± 13.16	47.66 ± 16.89	43.02 ± 17.86	4.75	0.001			0.010		0.004	
NonHDL (mg/dL)	91.84 ± 18.40	101.69 ± 24.92	99.79 ± 22.91	110.70 ± 25.22	5.69	0.001			<0.001			
Trig (mg/dL)	58.78 ± 22.09	72.05 ± 35.73	69.26 ± 30.03	84.23 ± 48.00	6.47	<0.001		0.002	0.001			
MBP (mmHg)	75.88 ± 9.40	80.19 ± 10.97	86.05 ± 9.27	86.47 ± 11.14	12.54	<0.001		<0.001	<0.001	0.016	0.006	
**Muscular fitness**												
BMI (kg/m^2^)	18.06 ± 1.20	19.65 ± 0.07	21.65 ± 0.31	25.83 ± 2.75	23.46	<0.001		0.011	<0.001		0.002	0.011
WC (cm)	62.84 ± 3.48	75.25 ± 6.72	70.43 ± 4.28	82.28 ± 7.10	14.82	<0.001	0.038		<0.001			0.025
FM (%)	19.45 ± 6.30	28.70 ± 2.55	23.95 ± 3.52	30.48 ± 5.73	2.17	0.137						
LDL (mg/dL)	55.00 ± 15.82	84.50 ± 12.02	67.00 ± 15.56	85.25 ± 36.58	2.16	0.139						
HDL (mg/dL)	80.25 ± 14.08	71.00 ± 8.49	66.75 ± 25.84	39.25 ± 4.99	2.39	0.112						
NonHDL (mg/dL)	72.88 ± 19.32	102.50 ± 16.26	88.50 ± 15.80	111.75 ± 36.83	2.66	0.089						
Trig (mg/dL)	88.88 ± 24.54	89.50 ± 21.92	106.75 ± 40.14	162.75 ± 72.26	0.808	0.510						
BMP (mmHg)	90.00 ± 19.94	87.50 ± 6.36	86.50 ± 11.62	92.00 ± 8.16	0.10	0.961						
**Physical fitness**												
BMI (kg/m^2^)	17.80 ± 1.24	18.96 ± 1.06	21.65 ± 0.31	25.83 ± 2.75	22.99	<0.001		0.013	<0.001		<0.001	0.012
WC (cm)	64.36 ± 3.34	66.28 ± 8.98	70.43 ± 4.28	82.28 ± 7.10	6.76	0.005			0.006		0.014	
FM (%)	19.16 ± 3.84	23.44 ± 8.90	23.95 ± 3.52	30.48 ± 5.73	1.67	0.218						
LDL (mg/dL)	58.60 ± 17.78	63.20 ± 22.20	67.00 ± 15.56	85.25 ± 36.58	1.04	0.404						
HDL (mg/dL)	86.60 ± 10.97	70.20 ± 10.52	66.75 ± 25.84	39.25 ± 4.99	3.43	0.047			0.040			
NonHDL (mg/dL)	77.20 ± 21.21	80.40 ± 24.79	88.50 ± 15.80	111.75 ± 36.83	1.57	0.240						
Trig (mg/dL)	92.60 ± 24.81	85.40 ± 22.95	106.75 ± 40.14	162.75 ± 72.26	0.81	0.510						
BMP (mmHg)	78.60 ± 11.70	100.40 ± 16.58	86.50 ± 11.62	92.00 ± 8.16	2.59	0.094						

Legend: FU = fat unfit; UU = unfat unfit; FF = fat fit; UF = unfat fit, BMI = body mass index; WC = waist circumference; MBP = median blood pressure; FM: fat mass; LDL: low-density lipoprotein; HDL: high-density lipoprotein; Trig: triglycerides; Symbols: >, < indicate statistical significance (*p* < 0.05) in pairwise mean comparisons using Bonferroni post hoc test.

## Data Availability

The data presented in this study are available on request from the corresponding author. The data are not publicly available due to privacy.

## References

[1] Kimm S., Obarzanek E. (2002). Childhood obesity: A new pandemic of the new millennium. Pediatrics.

[2] GBD 2015 Risk Factors Collaborators (2016). Global, regional, and national comparative risk assessment of 79 behavioural, environmental and occupational, and metabolic risks or clusters of risks, 1990–2015: A systematic analysis for the Global Burden of Disease Study 2015. Lancet.

[3] Jankowska A., Brzeziński M., Romanowicz-Sołtyszewska A., Szlagatys-Sidorkiewicz A. (2021). Metabolic Syndrome in Obese Children—Clinical Prevalence and Risk Factors. Int. J. Environ. Res. Public Health.

[4] Juonala M., Magnussen C.G., Berenson G.S., Venn A., Burns T.L., Sabin M.A., Raitakari O.T. (2011). Childhood adiposity, adult adiposity, and cardiovascular risk factors. N. Engl. J. Med..

[5] Correa-Rodríguez M., Ramírez-Vélez R., Correa-Bautista J.E., Castellanos-Vega R.D.P., Arias-Coronel F., González-Ruíz K., Alejandro H., Schmidt-RioValle J., González-Jiménez E. (2018). Association of Muscular Fitness and Body Fatness with Cardiometabolic Risk Factors: The FUPRECOL Study. Nutrients.

[6] Messina A., Monda M., Valenzano A., Messina G., Villano I., Moscatelli F., Cibelli G., Marsala G., Polito R., Ruberto M. (2018). Functional changes induced by orexin a and adiponectin on the sympathetic/parasympathetic balance. Front. Physiol..

[7] Freedman D.S., Kahn H.S., Mei Z., Grummer-Strawn L.M., Dietz W.H., Srinivasan S.R., Berenson G.S. (2007). Relation of body mass index and waist-to-height ratio to cardiovascular disease risk factors in children and adolescents: The Bogalusa Heart Study. Am. J. Clin. Nutr..

[8] Baker J.L., Olsen L.W., Sørensen T.I. (2007). Childhood body-mass index and the risk of coronary heart disease in adulthood. N. Engl. J. Med..

[9] Ahrens W., Moreno L.A., Mårild S., Molnár D., Siani A., Henauw S.D., Böhmann J., Günther K., Hadjigeorgiou C., Iacoviello L. (2014). Metabolic syndrome in young children: De fi nitions and results of the IDEFICS study. Int. J. Obes..

[10] Martínez-Vizcaíno V., Solera M., Salcedo F., Serrano S., Franquelo R., Sánchez M., Martínez P.M., Rodríguez-Artalejo F. (2010). Validity of a single-factor model underlying the metabolic syndrome in children. Cardiovasc. Metab. Risk.

[11] Torres-Costoso A., Garrido-Miguel M., Gracia-Marco L., López-Muñoz P., Reina-Gutiérrez S., Núñez de Arenas-Arroyo S., Martínez-Vizcaíno V. (2021). The “Fat but Fit” paradigm and bone health in young adults: A cluster analysis. Nutrients.

[12] Ortega F.B., Silventoinen K., Tynelius P., Rasmussen F. (2012). Muscular strength in male adolescents and premature death: Cohort study of one million participants. BMJ.

[13] Barry V.W., Baruth M., Beets M.W., Durstine J.L., Liu J., Blair S.N. (2014). Fitness vs. fatness on all-cause mortality: A meta-analysis. Prog. Cardiovasc. Dis..

[14] De Schutter A., Kachur S., Lavie C.J., Menezes A., Shum K.K., Bangalore S., Arena R., Milani R.V. (2018). Cardiac rehabilitation fitness changes and subsequent survival. Eur. Heart J. Qual. Care Clin. Outcomes.

[15] Elagizi A., Kachur S., Lavie C.J., Carbone S., Pandey A., Ortega F.B., Milani R.V. (2018). An Overview and Update on Obesity and the Obesity Paradox in Cardiovascular Diseases. Prog. Cardiovasc. Dis..

[16] Díez-Fernández A., Sánchez-López M., Mora-Rodriguez R., Notario-Pacheco B., Torrijos-Niño C., Martínez-Vizcaíno V. (2014). Obesity as a mediator of the influence of cardiorespiratory fitness on cardiometabolic risk: A mediation analysis. Diabetes Care.

[17] Garcia A., Agostinis C., Mota J., Santos R.M., Correa J.E., Ramírez R. (2017). Adiposity as a full mediator of the influence of cardiorespiratory fitness and inflammation in schoolchildren: The FUPRECOL Study. Nutr. Metab. Cardiovasc. Dis..

[18] Ortega F.B., Ruiz J.R., Castillo M.J., Sjöström M. (2008). Physical fitness in childhood and adolescence: A powerful marker of health. Int. J. Obes..

[19] Ortega F.B., Ruiz J.R., Labayen I., Lavie C.J., Blair S.N. (2017). The Fat but Fit paradox: What we know and don’t know about it. Br. J. Sports Med..

[20] García-Hermoso A., Cavero-Redondo I., Ramírez-Vélez R., Ruiz J., Ortega F.B., Lee D.-C., Martínez-Vizcaíno V. (2018). Muscular strength as a predictor of all-cause mortality in apparently healthy population: A systematic review and meta-analysis of data from approximately 2 million men and women. Arch. Phys. Med. Rehabil..

[21] Ruiz J.R., Sui X., Lobelo F., Morrow J.R., Jackson A.W., Sjöström M., Blair S.N. (2008). Association between muscular strength and mortality in men: Prospective cohort study. BMJ.

[22] Weisstaub G., Gonzalez Bravo M.A., García-Hermoso A., Salazar G., López-Gil J.F. (2022). Cross-sectional association between physical fitness and cardiometabolic risk in Chilean schoolchildren: The fat but fit paradox. Transl. Pediatr..

[23] Esparza-Ros F., Vaquero-Cristóbal R., Marfell-Jones M. (2019). International Standards for Anthropometric Assessment.

[24] Léger L.A., Mercier D., Gadoury C., Lambert J. (1988). The multistage 20 metre shuttle run test for aerobic fitness. J. Sport Sci..

[25] Ervin R.B., Fryar C.D., Wang C.Y., Miller I.M., Ogden C.L. (2014). Strength and body weight in US children and adolescents. Pediatrics.

[26] Stoner L., Pontzer H., Barone Gibbs B., Moore J.B., Castro N., Skidmore P., Lark S., Williams M.A., Hamlin M.J., Faulkner J. (2020). Fitness and Fatness Are Both Associated with Cardiometabolic Risk in Preadolescents. J. Pediatr..

[27] Sasayama K., Ochi E., Adachi M. (2015). Importance of both fatness and aerobic fitness on metabolic syndrome risk in Japanese children. PLoS ONE.

[28] Pozuelo-carrascosa D.P., Sánchez-lópez M., Cavero-redondo I., Torres-costoso A., Bermejo-cantarero A., Martínez-vizcaíno V. (2016). Obesity as a Mediator between Cardiorespiratory Fitness and Blood Pressure in Preschoolers. J. Pediatr..

[29] Kim J., Lee I., Lim S. (2017). Overweight or obesity in children aged 0 to 6 and the risk of adult metabolic-syndrome: A systematic review and meta-analysis. J. Clin. Nurs..

[30] Weihe P., Weihrauch-Blüher S. (2019). Metabolic syndrome in children and adolescents: Diagnostic criteria, therapeutic options and perspectives. Curr. Obes. Rep..

[31] Zimmet P., Alberti K., Kaufman F., Tajima N., Silink M., Arslanian S., Wong G., Bennett P., Shaw J., Caprio S. (2007). The metabolic syndrome in children and adolescents—An IDF consensus report. Pediatr. Diabetes.

[32] González-Gálvez N., Ribeiro J., Mota J. (2021). Metabolic syndrome and cardiorespiratory fitness in children and adolescents: The role of obesity as a mediator. J. Pediatr. Endocrinol. Metab..

[33] Suebsamran P., Pimpak T., Thani P., Chamnan P. (2018). The metabolic syndrome and health behaviors in school children aged 13–16 years in Ubon Ratchathani: UMeSIA project. Metab. Syndr. Relat. Disord..

[34] Buchan D., Young J., Boddy L., Baker J. (2014). Independent associations between cardiorespiratory fitness, waist circumference, BMI, and clustered cardiometabolic risk in adolescents. Am. J. Hum. Biol..

[35] Christodoulos A.D., Douda H.T., Tokmakidis S.P. (2012). Cardiorespiratory fitness, metabolic risk, and inflammation in children. Int. J. Pediatr..

[36] Bailey D.P., Savory L.A., Denton S.J., Kerr C.J. (2015). The association between cardiorespiratory fitness and cardiometabolic risk in children is mediated by abdominal adiposity: The HAPPY study. J. Phys. Act. Health.

[37] López-Martínez S., Sánchez-López M., Solera-Martinez M., Arias-Palencia N., Fuentes-Chacón R.M., Martínez-Vizcaíno V. (2013). Physical activity, fitness, and metabolic syndrome in young adults. Int. J. Sport Nutr. Exerc. Metab..

[38] Nyström C., Henriksson P., Martínez-Vizcaíno V., Medrano M., Cadenas-Sanchez C., Arias-Palencia N.M., Löf M., Ruiz J.R., Labayen I., Sánchez-López M. (2017). Does cardiorespiratory fitness attenuate the adverse effects of severe/morbid obesity on cardiometabolic risk and insulin resistance in Children? A pooled analysis. Diabetes Care.

[39] Eisenmann J.C., Welk G.J., Ihmels M., Dollman J. (2007). Fatness, fitness, and cardiovascular disease risk factors in children and adolescents. Med. Sci. Sports Exerc..

[40] García-Hermoso A., Carrillo H.A., González-Ruíz K., Vivas A., Triana-Reina H.R., Martínez-Torres J., Prieto-Benavidez D.H., Correa-Bautista J.E., Ramos-Sepúlveda J.A., Villa-González E. (2017). Fatness mediates the influence of muscular fitness on metabolic syndrome in Colombian collegiate students. PLoS ONE.

[41] Klimcakova E., Polak J., Moro C., Hejnova J., Majercik M., Viguerie N., Berlan M., Langin D., Stich V. (2006). Dynamic Strength Training Improves Insulin Sensitivity without Altering Plasma Levels and Gene Expression of Adipokines in Subcutaneous Adipose Tissue in Obese Men. J. Clin. Endocrinol. Metab..

[42] Holten M.K., Zacho M., Gaster M., Juel C., Wojtaszewski J.F.P., Dela F. (2004). Strength training increases insulin-mediated glucose uptake, GLUT4 content, and insulin signaling in skeletal muscle in patients with type 2 diabetes. Diabetes.

[43] Castro-Piñero J., Laurson K.R., Artero E.G., Ortega F.B., Labayen I., Ruperez A.I., Zaqout M., Manios Y., Vanhelst J., Marcos A. (2019). Muscle strength field-based tests to identify European adolescents at risk of metabolic syndrome: The HELENA study. J. Sci. Med. Sport.

[44] Thivel D., Ring-Dimitriou S., Weghuber D., Frelut M.-L., O’Malley G. (2016). Muscle Strength and Fitness in Pediatric Obesity: A Systematic Review from the European Childhood Obesity Group. Obes. Facts.

[45] Bull F.C., Al-Ansari S.S., Biddle S., Borodulin K., Buman M.P., Cardon G., Carty C., Chaput J.P., Chastin S., Chou R. (2020). World Health Organization 2020 guidelines on physical activity and sedentary behaviour. Br. J. Sports Med..

[46] Jurca R., Lamonte M.J., Barlow C.E., Kampert J.B., Church T.S., Blair S.N. (2005). Association of muscular strength with incidence of metabolic syndrome in men. Med. Sci. Sports Exerc..

[47] Andersen L.B., Lauersen J.B., Brønd J.C., Anderssen S.A., Sardinha L.B., Steene-johannessen J., McMurray R.G., Barros M.V.G., Kriemler S., Møller N.C. (2015). A new approach to define and diagnose cardiometabolic disorder in children. J. Diabetes Res..

[48] Albaladejo-Saura M., Vaquero-Cristóbal R., González-Gálvez N., Esparza-Ros F. (2021). Relationship between Biological Maturation, Physical Fitness, and Kinanthropometric Variables of Young Athletes: A Systematic Review and Meta-Analysis. Int. J. Environ. Res. Public Health.

[49] Albaladejo-Saura M., Vaquero-Cristóbal R., Esparza-Ros F. (2022). Métodos de estimación de la maduración biológica en deportistas en etapa de desarrollo y crecimiento: Revisión bibliográfica. Cult. Cienc. Y Deporte.

[50] Tomkinson G.R., Carver K.D., Atkinson F., Daniell N.D., Lewis L.K. (2018). European normative values for physical fitness in children and adolescents aged 9–17 years: Results from 2 779 165 Eurofit performances representing 30 countries. Br. J. Sports Med..

[51] Reche-García C., Hernández Morante J.J., Trujillo Santana J.T., González Cisneros C.A., Romero Romero J., Ortín Montero F.J. (2022). Bienestar psicológico de deportistas adolescentes mexicanos confinados por la pandemia del COVID-19. Cult. Cienc. Y Deporte.

[52] Berrios B., Latorre P., Salas J., Pantoja A. (2022). Effect of physical activity and fitness on executive functions and academic performance in children of elementary school. A systematic review (Efectos de la actividad física y condición física sobre funciones ejecutivas y rendimiento académico en niños de Educación Primaria. Una revisión sistemática). Cult. Cienc. Deporte.

